# Supplementary material for: Process reveals structure: How a network is traversed mediates expectations about its architecture
Source: Sci Rep. 2017 Oct 6;7:12733. doi: 10.1038/s41598-017-12876-5 (PMC5630604; doi:10.1038/s41598-017-12876-5)
Supplement: Supplementary file 1 — Supplementary Information [file 41598_2017_12876_MOESM1_ESM.doc]

**TITLE: Process reveals structure: How a network is traversed mediates expectations about its architecture**

AUTHORS: Elisabeth A. Karuza^1*^, Ari E. Kahn^2,3^, Sharon L. Thompson-Schill^1,4^, & Danielle S. Bassett^3,5^

AFFILIATIONS:

^1^Department of Psychology, University of Pennsylvania, Philadelphia, PA 19104 USA

^2^Department of Neuroscience, University of Pennsylvania, Philadelphia, PA 19104 USA

^3^Department of Bioengineering, University of Pennsylvania, Philadelphia, PA 19104 USA

^4^Department of Neurology, University of Pennsylvania, Philadelphia, PA 19104 USA

^5^Department of Electrical and Systems Engineering, University of Pennsylvania, Philadelphia, PA 19104 USA

*CORRESPONDING AUTHOR:

Elisabeth A. Karuza

[ekaruza@sas.upenn.edu](mailto:ekaruza@sas.upenn.edu)

**Supplementary Information**


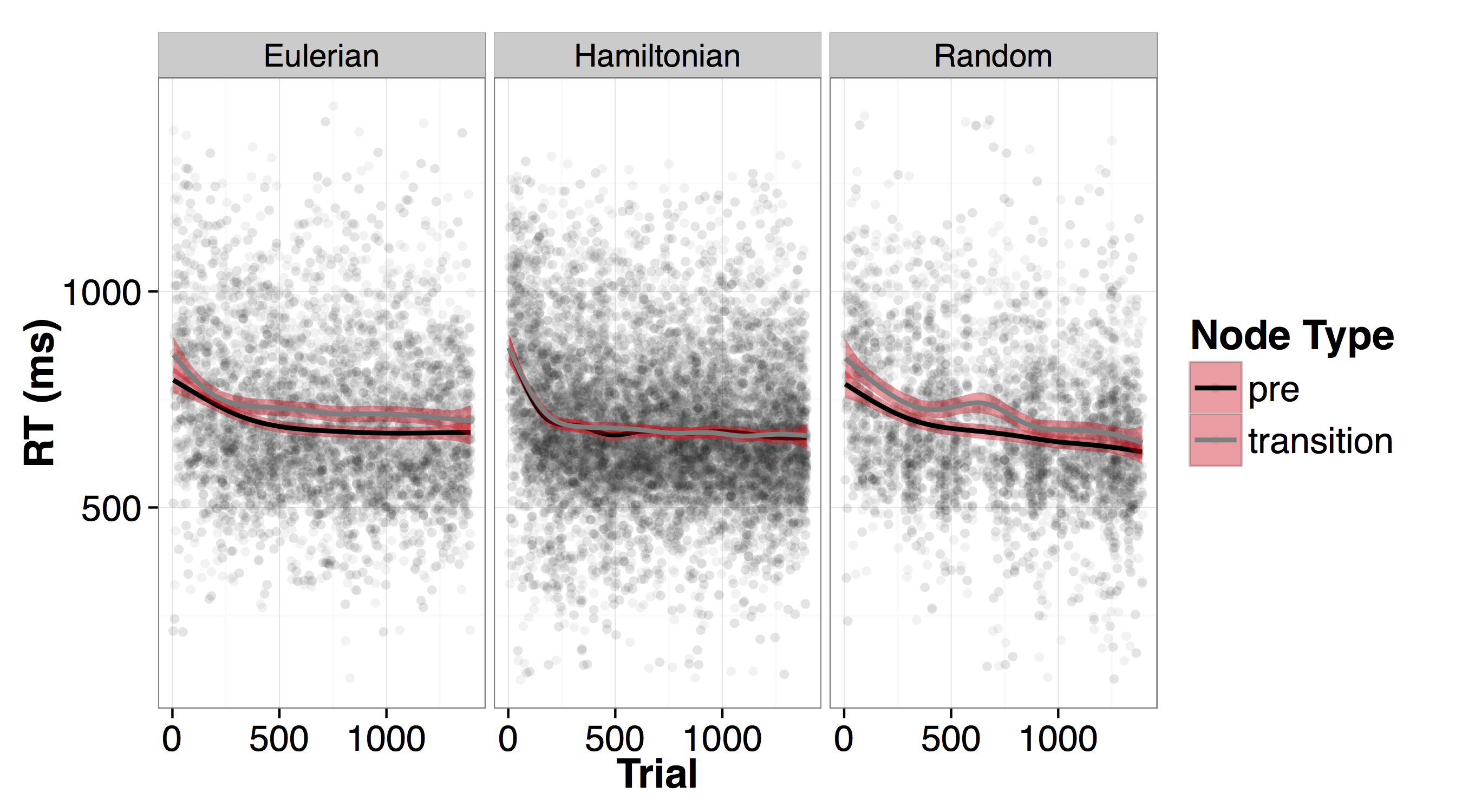
*Section 1. Indexing Response Patterns Over Time*

*Figure S1. Reaction time plotted as a function of trial number, smoothed with a generalized additive function. RTs were increasingly facilitated for both pre-transition (black) and transition (grey) nodes. Panels correspond to each experimental condition (i.e., walk type).*

*
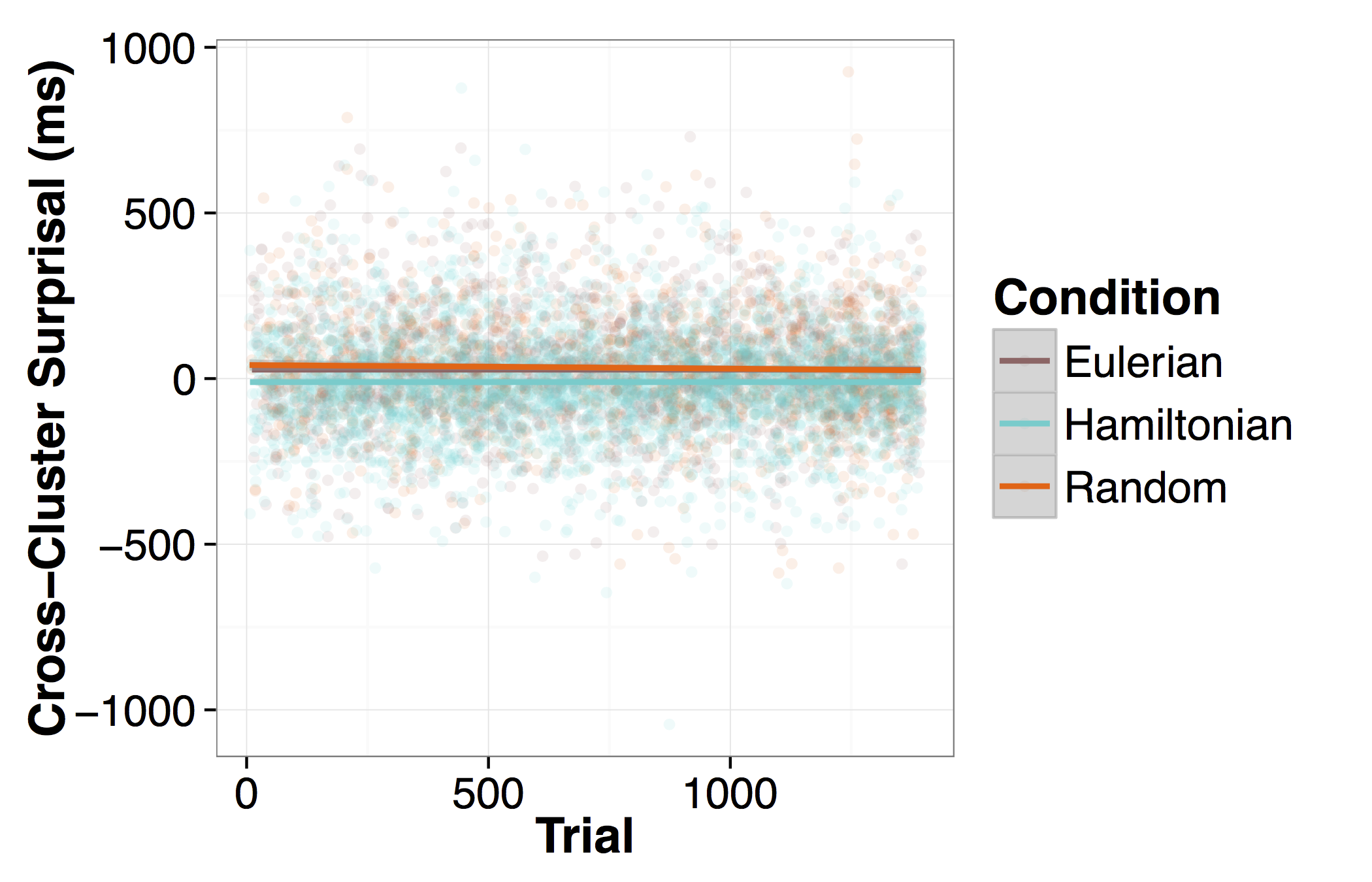
*

*Figure S2. Cross-community surprisal effects (RTs for transition – pre-transition nodes) plotted as a function of trial number, smoothed with a generalized additive function.*

*Section 2. Alternative Priming Accounts*

Because there are many potential ways to account for low-level perceptual priming effects, we present here an alternative method based on a moving time window of interest. We also note that our confound regressors may not fully account for the full extent of low-level perceptual priming. RTs from the Random and Hamiltonian conditions were again regressed onto all main effects and interactions of Node Type (pre *versus* transition), Condition and Trial Number, plus the addition of three new binary priming predictors that captured whether a given node had been seen in the previous 2–3 trials, 4–6 trials, or 7–12 trials. This repetition priming model included a random intercept for participant and by-participant random slopes for Trial, Node Type, and the interaction between Trial and Node Type. The full table of results is presented below (Table S1). Though main effects of priming for each of the three predictors are clearly observed, all previous significant effects are maintained: main effects of Node Type and Trial, as well as Node Type*Condition and Condition*Trial interactions. As in our other priming analysis, we again verified via model comparison that this full model provided a superior fit to the data relative to a model that did not include main effects or interactions of Condition and Node Type (i.e., that only included the three repetition priming predictors and Trial Number). Indeed, a comparison of the log likelihood ratio between the two models, after they were fit using a Maximum Likelihood procedure, was significant (χ2 = 25.74, p = 0.0002).

**Table S1. Coefficients (and corresponding t-values and p-values) for each predictor in a model comparing the Random and Hamiltonian conditions.** Specifically, we examine the effect of Node Type (pre-transition *versus* transition), Condition (Random *versus* Hamiltonian) and Trial on RTs from the exposure phase. This model now also includes three new priming predictors (listed below as Lag2–3, Lag4–6, and Lag7–12). Significant values (determined using the Sattherwaite approximation and corresponding to p < 0.05) are shown in boldface type.

| **Predictor** | **Coefficient** | **T-value** |  | **P-value** |
| --- | --- | --- | --- | --- |
| Alternative priming model |  |  |  |  |
| **Node Type (pre v. transition)** | **7.66** | **4.03** |  | **<0.001** |
| Condition (Random v. Hamiltonian) | –9.71 | –0.89 |  | >0.250 |
| **Trial** | **–31.15** | **–7.69** |  | **<0.001** |
| **Lag2–3** | **–12.43** | **–3.76** |  | **<0.001** |
| **Lag4–6** | **–9.30** | **–3.79** |  | **<0.001** |
| **Lag7–12** | **–8.50** | **–3.42** |  | **<0.001** |
| **Node Type*Condition** | **–5.96** | **–3.24** |  | **0.001** |
| Node Type*Trial | –2.43 | –1.54 |  | 0.125 |
| **Condition*Trial** | **8.26** | **2.04** |  | **0.048** |
| Node Type*Condition*Trial | 1.02 | 0.65 |  | >0.250 |

*Section 3. Post-Exposure Measures*

**Methods**

**Materials**

**Segmentation sequence.** The segmentation sequence was identical across all three conditions. It consisted of groupings of 15 images that alternated between a random walk and one fixed Hamiltonian path. The Hamiltonian path was entered at a randomly selected node adjacent to the terminal node of the random walk, and included both backward and forward transversals. We opted to use the random/ Hamiltonian path combinations (as opposed to separate segmentation sequences paralleling each exposure sequence) in order to make a direct connection with prior work (Schapiro et al., 2013). This test format also enabled us to probe whether any learning effects from the exposure phase might generalize during exposure to a novel walk sequence. As in the exposure phase, each image was presented for 1500 ms with no interstimulus interval. The entire sequence contained 600 items.

**Odd-man out stimuli.** Items in the odd-man out task consisted of a triplet of images spanning a community boundary. Two of those images belonged to the same community (one was a boundary node), while the third was a boundary node outside that community (but sharing an edge with the other boundary node in the trio). All three images were presented simultaneously. The post-test phase consisted of 18 trials corresponding to all possible orderings of unique 3 test triplets (nodes 1, 13, 15; nodes 3, 5, 6; nodes 8, 10, 11; Fig.1).

**Procedure**

**Segmentation phase.** During the segmentation phase, participants were instructed as follows: “You have only one task: you’ll see a stream of the same images presented in their regular orientation, we want you to press the spacebar at times in the sequence that you feel are natural breaking points. If you’re not sure, go with your gut feeling. Try to make your responses as quickly and accurately as possible.” They again completed a quiz to ensure their understanding. Participants received no performance bonus for their demonstrated ability to segment the stream. Regardless of condition they received one of two segmentation lists, counterbalanced with one of two exposure lists.

**Odd-man out phase.** In the final phase of the experiment, participants completed an odd-man out task. Trial order was randomized by subject and again, participants received no performance bonus. They were instructed as follows: “The stream of images you just saw adhered to a pattern. In other words, some of the images you saw “went together.” We want to see how well you learned that pattern. For each trial, you’ll be presented with three images in random order. We’re interested in whether or not you can pick the single image that DOESN’T belong based on what you just saw in the previous two phases of the experiment.” Trial duration was unlimited, and the start of each subsequent trial was triggered by a button press.

**Analyses**

**Data exclusions.** In the segmentation phase, we compared parsing probabilities (Fig. S3) only for those participants who surpassed the following predetermined exclusion criteria: fewer than 5 button presses per 600 trials or greater than 10 button presses in a row. These criteria resulted in exclusion of over 20% of participants (13/60). In general, online participants exhibited lower likelihood of a parse (0.159 at community transitions and 0.135 elsewhere) compared to participants in Schapiro et al., who tended toward parsing performance upwards of 0.3 at community transitions. Lowered data quality on these post-exposure measures prevents us from drawing strong conclusions about the explicit expression of graph knowledge. We stress here that financial incentives based on performance, ideally on an orthogonal cover task as in the present experiment’s exposure phase, are essential when collecting data in an on-line marketplace.

**Results**

Participants were no more likely to segment the stream at a community boundary relative to elsewhere in the sequence (Mean difference Random: 0.029, s.d. = 0.094, *t(*16) = 1.233, *p* = 0.235; Mean difference Eulerian: 0.016, s.d. = 0.061, *t(*13) = 0.990, *p* = 0.340; Mean difference Hamiltonian: 0.024, s.d. = 0.108, *t(*14) = 0.862, *p* = 0.403). In the odd-man out judgment, participants were simultaneously presented with three images, two of which were drawn from the same community. They were asked to indicate which image “did not belong” with the others. Performance did not differ significantly from chance in any condition (Random mean = 0.267, s.d. = 0.182; versus chance, *t(*19) = 1.641, *P* = 0.1173; Eulerian mean = 0.313, s.d. = 0.184; versus chance, *t(*18) = 0.486, *P* = 0.633; Hamiltonian mean = 0.311, s.d. = 0.050; versus chance, *t(*19) = 0.448, *P* = 0.659).

Figure S3. Segmentation task performance. Across all experimental conditions (Eulerian, Hamiltonian, Random), participants were no more likely to parse a sequence at a community transition relative to any other node. The segmentation data presented here excludes participants who failed to comply with task directions for this portion of the experiment (remaining *N* = 47).
